# Potential Anticancer Properties of Grape Antioxidants

**DOI:** 10.1155/2012/803294

**Published:** 2012-08-07

**Authors:** Kequan Zhou, Julian J. Raffoul

**Affiliations:** ^1^Department of Nutrition and Food Science, Wayne State University, Detroit, MI 48202, USA; ^2^Department of Medicine, Emory University School of Medicine, Atlanta, GA 30322, USA

## Abstract

Dietary intake of foods rich in antioxidant properties is suggested to be cancer protective. Foods rich in antioxidant properties include grape (*Vitis vinifera*), one of the world's largest fruit crops and most commonly consumed fruits in the world. The composition and cancer-protective effects of major phenolic antioxidants in grape skin and seed extracts are discussed in this review. Grape skin and seed extracts exert strong free radical scavenging and chelating activities and inhibit lipid oxidation in various food and cell models *in vitro*. The use of grape antioxidants are promising against a broad range of cancer cells by targeting epidermal growth factor receptor (EGFR) and its downstream pathways, inhibiting over-expression of COX-2 and prostaglandin E2 receptors, or modifying estrogen receptor pathways, resulting in cell cycle arrest and apoptosis. Interestingly, some of these activities were also demonstrated in animal models. However, *in vivo* studies have demonstrated inconsistent antioxidant efficacy. Nonetheless, a growing body of evidence from human clinical trials has demonstrated that consumption of grape, wine and grape juice exerts many health-promoting and possible anti-cancer effects. Thus, grape skin and seed extracts have great potential in cancer prevention and further investigation into this exciting field is warranted.

## 1. Introduction

Grape (*Vitis vinifera*) is one of the world's largest fruit crops. Grape is also one of the most commonly consumed fruits in the world both as fresh fruit (table grape) and processed fruit (wine, grape juice, molasses, and raisins) [1, 2]. There are greater than one hundred grape species which are divided into 2 subgenera: *Euvitis and Muscadinia*. Most of the species are in *Euvitis* subgenera [3]. Commercially cultivated grapes are usually classified as table or wine grapes depending on their intended method of consumption [4]. Table grapes usually bear large, seedless fruit berries with relatively thin skin, whereas wine grapes are smaller with thick skins [5]. Grapes are known to contain substantial amount of simple sugars especially glucose and fructose. For instance, wine grapes usually contain 19% or higher sugar content by fresh weight [6]. A growing body of epidemiological studies and randomized controlled human trials have associated the consumption of grapes, wine, and grape juice with a wide variety of health-promoting effects particularly the reduced risk of cardiovascular diseases, type-2 diabetes, certain types of cancers, and other chronic complications [7–13].

## 2. Phenolic Antioxidants in Grapes

The beneficial effects of grape and relevant grape-derived food products are believed to be related to a variety of bioactive components in grapes [14–16]. One major group of these components is phenolic antioxidants typically including anthocyanins, catechins, resveratrol, phenolic acids, and procyanidins [17]. Based on our analysis, 100 grams of fresh grapes contain 63–182 mg of the phenolic compounds [18]. Flavonoids constitute the majority of phenolic compounds (65–76%) in grapes. In red grapes, anthocyanins are the major group of the flavonoids [18]. Most of grape phenolic antioxidants are distributed in grape skins or seeds [19]. For instance, resveratrols, antochyanins, and catechins are concentrated in the skin part, while procyanidins are concentrated in grape seeds [20]. The commercial grape skin or seed extracts are made from grape pomace which is typically regarded as a waste byproduct generated in the lucrative winemaking industry [21]. Large amounts of this byproduct accumulate annually which leads to a waste-management issue [22]. It is estimated that the harvested grapes will generate approximately 20% of grape pomace. However, uses of grape pomace are limited but have been recycled as organic fertilizers, manure, and animal feed [23]. Because grape skins and seeds are the predominant constituents in the pomace, this biomass is a rich source of phenolic antioxidants [20, 21]. We have previously extracted phenolic compounds from the skins of fresh Norton grapes which are wine grapes with unusual small sizes. The Norton grape skin contains 215.6 mg phenolic compounds per gram of the extract [24]. The most abundant components in grape skin are those flavonoids. The concentration of catechin and epicatechin is 8.71 and 3.45 mg per gram of the Norton skin extract [24]. Grape skin also contains a substantial amount of phenolic acids such as gallic acid, ferulic acid, caffeic acid, syringic acid, and p-coumaric acid with some being bound with sugars. Resveratrol is one of the most prominent bioactive components in grapes. Although resveratrol is predominantly contained in the grape skin, its concentration is only 0.21 mg per gram of the extract based on our HPLC analysis [24].

It is estimated that approximately 60–70% of grape polyphenols exist in grape seeds [25]. In contrast to grape skins, grape seeds contain a main unique group of phenolic compounds, procyanidins, which are flavan-3-ol derivatives and are colorless in the pure state [26]. Oligomeric and polymeric procyanidins in grape seeds possess a broad spectrum of pharmacological, medicinal, and therapeutic properties and are one of the most potent natural antioxidants [27–30]. They can be extracted during the latter stages of wine-making and are believed to contribute to color stability and organoleptic properties of wine [4–6]. In recent years, these proanthocyanidin compounds have extracted and purified from grape seeds and have become a common nutritional supplement. On the basis of structural characteristics, grape seed proanthocyanidins belong to condensed tannins. They mostly consist of (+) catechin and (−) epicatechin units, linked by C–4–C–8 or C–4–C–6 bonds and sometimes esterified by gallic acid on the epicatechin moiety(ies) [26]. These proanthocyanidins are the oligomer and polymer of flavan-3-ol with an average degree of polymerization (DP) ranging from 2 to >15 and an average molecular mass ranging from 578 to >5000 Da [1]. The structures of grape dimeric and trimeric proanthocyanidins have been recently elucidated [26]. The proanthocyanidin content in grape seeds is highly dependent on their varieties and extraction procedures. In general, the galloylated procyanidins are present in considerably lower concentrations than the nongalloylated ones [31], and higher molecular weight polymers constitute the majority of proanthocyanidins in grape seeds [26].

## 3. Antioxidant Properties of Grape Phenolic Compounds

Antioxidant activities of grape phenolic compounds have been extensively investigated *in vitro* and *in vivo*. Studies from us and others showed that grape skin, seed, and pomace extracts possess potent free radical scavenging activities using oxygen radical absorbance capacity (ORAC), 2,2-diphenyl-1-picrylhydrazyl (DPPH), and 2,2′-azino-bis(3-ethylbenzothiazoline-6-sulphonic acid) (ABTS). Assays and some complex phenolics also show significant chelating activity on transition metal ions which are strong promoters of lipid peroxidation [18, 24, 27, 32]. The antioxidant activities of grape phenolics have also been demonstrated in various model systems such as protecting low-density lipoprotein (LDL) against oxidation brought about by Cu^2+^, oxygen-centered radical-generating AAPH, or peroxynitrite-generating SIN-1 *in vitro* systems, preventing spleen cells from DNA damage induced by hydrogen peroxide (H_2_O_2_), and reducing oxidative stress in PC12 cells induced by addition of Fe^2+^ and *t*-butyl hydroperoxide [33–35]. However, the *in vivo* studies examining antioxidant activity of grape extracts have shown inconsistent results. Some studies showed that dietary intake of grape antioxidants helps to prevent lipid oxidation and inhibit the production of reactive oxygen species (ROS). For instance, dietary supplementation of grape seed extract (600 mg/day) for 4 weeks was shown to reduce oxidative stress and improve glutathione (GSH)/oxidized glutathione (GSSG) and total antioxidant status (TAOS) in a double-blinded randomized crossover human trial [9]. Another study also demonstrated that grape seed extract supplementation (2 × 300 mg/day) improved plasma antioxidant capacity in the high-cholesterol human subjects [36]. While other studies showed that dietary supplementation of grape juice, grape skin, or grape seed extracts exhibits either only a moderate antioxidative effect [11, 37, 38] or a neutral effect in animals and humans [39–41]. We have recently found that 3-month dietary supplementation of grape skin and grape pomace antioxidant extracts (0.2% in diet, equivalent to equivalent to approximately 960 mg GSE/day for humans) showed no effects on oxidative stress in diet-induced obesity mice [41, 42]. These results are consolidated by a recent report which showed that dietary supplementation of grape skin extract (1170 mg/day) had no significant effect on antioxidant enzymes including superoxide dismutase or catalase and also had no effect on 2-aminoadipic semialdehyde (AAS) residues, a plasma protein oxidation product, or on malondialdehyde in plasma or in LDL, markers of lipoprotein oxidation in physically active individuals [43]. This inconsistency may be related to the low absorption of grape phenolics since the absorption rate of polyphenol antioxidants is generally less than 1% [44].

## 4. Anticancer Properties of Grape Phenolic**** Compounds

Grape antioxidants have drawn an increased attention for their potential anticancer effects. A number of studies suggest that the high consumption of grape components could be associated with the reduced risk of certain cancers such as breast cancer and colon cancer [45–47]. The anticancer effects of grape antioxidants have been demonstrated in *in vitro* and *in vivo* models [48–52]. Grape antioxidants have been shown to induce cell cycle arrest and apoptosis in cancer cells [53] as well as prevent carcinogenesis and cancer progression in rodent models [54, 55]. Considering the diversity of grape antioxidants, it is very likely that these compounds are to exert potential anticancer activity by acting on multiple cellular events associated with tumor initiation, promotion, and progression. Proposed mechanisms of potential anticancer effects of grape antioxidants include antioxidant, anti-inflammatory, and antiproliferative activities [56]. Grape antioxidants could act as free radicals scavengers, and chelating agents help to reduce physiological reactive oxygen species (ROS) [57]. ROS is known as an important mediator of apoptosis since initiation and regulation of apoptosis is associated with modifications in the oxidative environment [58]. Study has shown that dietary intake of grape antioxidants reduced rat mucosal apoptosis via modulation of both mitochondrial and cytosolic antioxidant enzyme systems together with an increase in cellular glutathione (GSH): glutathione disulfide (GSSG) ratio, protecting normal colonic mucosa from reactive oxygen species (ROS) attack [25, 59]. Grape antioxidants also exert anti-inflammatory activity which is believed to be associated with their chemopreventive effects [25, 41, 42]. Cancer chemopreventive agents include nonsteroidal anti-inflammatory drugs (NSAIDs) such as indomethacin, piroxicam, and sulindac, all of which inhibit cyclooxygenase (COX) [60]. This inhibitory activity is relevant to cancer chemoprevention because COX catalyzes the conversion of arachidonic acid to proinflammatory substances such as prostaglandins, which can stimulate tumor cell growth and suppress immune surveillance [60, 61]. Moreover, COX may activate carcinogens to more reactive substances that cause genetic damage [62]. A number of studies have demonstrated the inhibitory effects of whole grape extracts, individual grape antioxidants, or their mixture on COX activity and gene expression [63–65]. In addition, a study found that grape antioxidants exert an antitumor activity partially related to their immunopotentiating activities through the enhancements of lymphocyte proliferation, NK cell cytotoxicity, CD4+/CD8+ ratio, IL-2, and IFN-*γ* productions [66].

Grape antioxidants are also shown to modify estrogen receptor (ER) and are therefore especially relevant for gynecological cancers such as breast cancer [67]. For example, some grape antioxidants such as resveratrol, quercetin, and catechin exhibit both estrogenic and antiestrogenic effects due to their structural similarity to the steroid hormone estrogen [67, 68]. Resveratrol was reported to bind ER*β* and ER*α* with comparable affinity but that the estrogen agonist activity of resveratrol was greater with ER*β* than with ER*α* [69]. The binding affinity of grape skin anthocyanidins (anthocyanin aglycones) to ER*α* was 10,000- to 20,000-fold lower than that of the endogenous estrogen estradiol and these compounds at treatment levels of 10–20 *μ*mols/L on MCF-7 cells exhibited a weak but statistically significant estrogenic activity [70]. However, in combination treatments with estradiol, three grape anthocyanidins showed antiestrogenic activity, which could be potentially explained by competition for the estrogen receptor and lower intrinsic activity of the phytoestrogens than estradiol [70]. It has been suggested that the number of hydroxy groups in grape pigments had a substantial effect on the estrogen activity. The presence of up to two hydroxy groups in the B-ring of the molecular structure decreased the affinity of the anthocyanidins to the ER*α* [71]. Grape anthocyanidins showed estrogen-inducible cell proliferation in MCF-7 breast cancer cell line but not in the receptor-negative MDA-MB-231 cell line. The fact that 4-hydroxytamoxifen, the receptor antagonist, can block the anthocyanidins-induced cell proliferation and combination treatments of anthocyanidins with estradiol-reduced proliferative activity of estradiol strongly suggest that the estrogenic activity of certain grape is relevant to its beneficial activity against estrogen-dependent cancers [70].

Epidermal growth factor receptor (EGFR) is a type I tyrosine kinase receptor belonging to a family of receptors that also includes HER2, HER3, and HER4. Aberrant EGFR activation, mediated primarily through changes in gene amplification and autocrine stimulation, appears to be a key factor in tumorigenesis, as well as an essential driving force for the aggressive growth behavior of cancer cells [72]. Grape antioxidants have been shown to inhibit expression of epidermal growth factor receptor (EGFR) in head and neck squamous cell carcinoma (HNSCC) cells which also caused an inhibition of the phosphorylation of extracellular signal-regulated kinase (ERK1/2), the highly conserved Ras/mitogen-activated protein kinase (MAPK)-dependent pathway (one of EGFR major downstream pathways) [57]. This effect is supported by another study that found that grape seed antioxidants inhibited constitutive activation of MAPK/ERK1/2 and MAPK/p38 in MDA-MB-468 cells [73]. The effects of grape antioxidants on cell cycle arrest are reported to be involved in promoting the expression of p21(Cip1)/p27(Kip1) protein G1-phase arrest [74]. Grape antioxidants can also target the transcription factor nuclear factor kappa B (NF-*κ*B) by inhibiting its DNA-binding capacity to inhibit cancer cell invasion [75]. Recent *in vitro* and *in vivo* studies on potential anticancer properties of grape antioxidants are discussed as follows.

## 5. *In Vitro* Anticancer Properties of Grape ****Phenolic Compounds

Grape antioxidants especially grape seed proanthocyanidins (GSPs) show very promising inhibitory effects on a variety of cancer cells. Non-small-cell lung cancer (NSCLC) represents approximately 80% of total lung cancer cases, the leading cause of cancer-related deaths. GSPs have been shown to induce apoptosis of NSCLC cells: A549 and H1299, which are mediated through increased expression of proapoptotic protein Bax, decreased expression of antiapoptotic proteins Bcl2 and Bcl-xl, disruption of mitochondrial membrane potential, and activation of caspases 9, 3, and poly(ADP-ribose) polymerase (PARP) [76]. Overexpression of COX-2 and prostaglandins (PG) is linked to a wide variety of human cancers. *In vitro* treatment of NSCLC cells (A549, H1299, H460, H226, and H157) with GSPs resulted in significant growth inhibition and induction of apoptosis, which were associated with the inhibitory effects of GSPs on the overexpression of COX-2 and prostaglandin (PG) E2 receptors (EP1 and EP4) in these cells [77].

Grape seed extract (GSE) was also found to selectively inhibit the growth and cause cell cycle arrest and apoptotic death in both Detroit 562 and FaDu HNSCC cells by activating DNA damage checkpoint cascade, including ataxia telangiectasia mutated/ataxia telangiectasia-Rad3-related-checkpoint kinase 1/2-cell division cycle 25C as well as caspases 8, 9, and 3 [78]. In addition, GSE-caused accumulation of intracellular ROS was identified as a major mechanism of its effect for growth inhibition, DNA damage and apoptosis, which was remarkably reversed by antioxidant N-acetylcysteine [78]. GSPs have recently shown to concentration-dependent inhibit human cutaneous HNSCC cell invasion, which was associated with a reduction in the levels of epidermal growth factor receptor (EGFR) and the inhibition of the phosphorylation of ERK1/2, a member of mitogen-activated protein kinase family [57]. Additionally, inhibition of human cutaneous HNSCC cell invasion by GSPs was associated with reversal of epithelial-to-mesenchymal transition (EMT) process, which resulted in an increase in the levels of epithelial biomarker (E-cadherin) while loss of mesenchymal biomarkers (vimentin, fibronectin, and N-cadherin) in cells [57]. These data suggest a potential for GSPs to be developed and used for the prevention of invasion/metastasis of HNSCC cells.

Melanoma is the leading cause of death from skin disease, and treatment of human melanoma A375 and Hs294t cells with GSPs resulted in a concentration-dependent inhibition of invasion or cell migration of these cells, which was related to a significant reduction in the levels of COX-2 expression and PGE(2) production [79]. GSPs *in vitro* are also effective on oral squamous cell carcinoma (OSCC). OEC-M1 cells lead to cell cycle arrest by increasing the expression of p21(Cip1)/p27(Kip1) protein without functioning mitochondria-mediated apoptosis, whereas GSP on SCC-25 cells inhibits cell proliferation via both G1-phase arrest and mitochondria-mediated apoptosis in a dose-dependent manner as a result of alterations of Bcl-2 [74]. Moreover, GSP can inhibit the migration and invasion of both cells, which are associated with the suppression of matrix metalloproteinases (MMPs), MMP-2, and MMP-9 [74].

Studies investigating the effect of grape products on breast cancer also show promising results. A polyphenolic fraction isolated from grape seeds that is rich in procyanidins inhibits constitutive activation of MAPK/ERK1/2 and MAPK/p38 and causes an induction of CDKI Cip1/p21 and a decrease in CDK4 in MDA-MB-468 cells [73]. Constitutive activation of ERK1/2 pathway has been shown to be associated with human breast carcinomas and derived cell lines for uncontrolled growth [80, 81]. These effects of GSP result in a G1 arrest in cell cycle, followed by an irreversible inhibition of cell growth [73]. The activity is supported by another study which found that GSE upregulates p21 (Cip1) through redox-mediated activation of ERK1/2 pathway and posttranscriptional regulation leading to cell cycle arrest in colon carcinoma HT29 cells [82].

GSE is also protective against prostate cancer which was shown to inhibit histone acetyltransferases (HATs) in LNCaP cells, leading to decreased androgen-receptor- (AR-) mediated transcription and cancer cell growth [83]. In addition, GSE can downregulate urokinase plasminogen activator (uPA) and DNA-binding activity of the transcription factor nuclear factor kappa B (NF-*κ*B) in highly metastatic androgen-independent PC3 prostate cancer cells and therefore inhibits cell invasion [75]. Study found that procyanidins from wild grape seeds can regulate ARE-mediated enzyme expression via Nrf2 coupled with p38 and PI3K/Akt pathway in HepG2 cells and could be used as a potential natural chemopreventive agent through Nrf2/ARE-mediated phase II detoxifying/antioxidant enzymes induction via p38 and PI3K/Akt pathway [84]. Growth of certain colon cancer cells is also inhibited by GSE which exerts both antiproliferative and apoptotic effects on Caco2 and HCT-8 colon cancer cells, and its inhibitive effects were stronger than isolated procyanidins, suggesting a potential additive or synergistic effect among the grape seed components [85]. Another study found that the combination of resveratrol, a prominent grape skin component, and grape seed extract induces much more pronounced apoptosis in colon cancer cells, which is strongly correlated with p53 levels and Bax : Bcl-2 ratio [86].

## 6. *In Vivo* Anticancer Properties of Grape ****Phenolic Compounds

Most *in vivo* studies of anticancer properties of grape components focused on GSE or proanthocyanidin fraction of GSE (e.g., GSPs). In 1999, Agarwal and his colleagues conducted a thorough review on *in vivo* efficacy and potential working mechanisms of GSE and grape-based products against a variety of cancers [87]. Therefore, in this paper we discussed the *in vivo* studies that were conducted since 1999. Dietary intake of GSE (0.2% GSE wt/wt in diet) decreased HNSCC Detroit 562 and FaDu xenograft tumor growth by 67 and 65% (*P* < 0.001), respectively. Xenografts from GSE-fed groups showed decreased proliferation but increased DNA damage and apoptosis [78]. However, the exact molecular mechanisms are remained to be elucidated. Administration of 50, 100, or 200 mg GSPs/kg body weight of mice by oral gavage (5 d/week) markedly inhibited the growth of NSCLC A549 and H1299 lung tumor xenografts in athymic nude mice, which was associated with the induction of apoptotic cell death, increased expression of Bax, reduced expression of antiapoptotic proteins, and activation of caspase-3 in tumor xenograft cells [76], suggesting a consistency between the observations of *in vitro* and *in vivo* studies. The growth-inhibitory effect of dietary GSPs (0.5%, w/w) was also shown on the NSCLC xenografts, and the inhibition was associated with the inhibition of COX-2, PGE(2), and PGE(2) receptors (EP1, EP3, and EP4) in tumors [77]. GSE may also be effective in the prevention of certain types of cancers. A recent study showed that dietary supplementation of grape seed extract (0.25 or 0.5% (w/w) caused strong chemopreventive efficacy in Fischer 344 rats against azoxymethane- (AOM-)induced aberrant crypt foci (ACF) formation (as much as 60% reduction in number of ACF and 66% reduction in crypt multiplicity) [88].

The literature search on various databases found only one human trial on grape products and cancer treatment that examined the role of grape products specifically grape seed proanthocyanidin extract in patients with breast induration following radiotherapy for breast cancer and found no significant effect. With respect to preventive effect, a human study showed that 8-week dietary supplementation of grape juice (480 mL/day) reduced lymphocyte DNA damage by reducing the formation of reactive oxygen species by as much as 15% [81], suggesting that a potential anticarcinogenic role for grape products by providing antioxidant protection.

In summary, with respect to cancer prevention and treatment, grape seed extracts or its proanthocyanidins have received most investigation for their potential anticancer activities. Some studies show very promising potential of GSE as an anticancer agent. However, most available reports are focusing on their *in vitro* activities and mechanisms. Since the absorption of these phenolic compounds is limited, the dose responses of GSE and its components need to be determined in animals and humans as well as its *in vivo* mechanisms. The potential use of grape skin and seed extracts in cancer prevention has great potential, and further investigation into this exciting field is warranted.
